# Genetic Polymorphism in the* RYR1* C6487T Is Associated with Severity of Hypospadias in Chinese Han Children

**DOI:** 10.1155/2018/7397839

**Published:** 2018-06-20

**Authors:** Haiyan Zhang, Zhuo Zhang, Linpei Jia, Wei Ji, Hai Li

**Affiliations:** ^1^Department of Gastrointestinal Surgery, China-Japan Union Hospital of Jilin University, Changchun 130033, China; ^2^Department of Urology, China-Japan Union Hospital of Jilin University, Changchun 130033, China; ^3^Department of Nephrology, Xuanwu Hospital of Capital Medical University, Beijing 100053, China; ^4^Department of Vascular Surgery, Jilin Provincial People's Hospital, Changchun 130000, China

## Abstract

**Objective:**

Hypospadias is a common congenital malformation of the male external genitalia. Most cases have an unknown etiology, which is probably a mix of monogenic and multifactorial forms, implicating both genetic and environmental factors. Ryanodine receptor 1 (*RYR1*) mutations are a common cause of congenital diseases associated with both dominant and recessive inheritance in humans. Herein, we evaluated the correlations of* RYR1* C6487T polymorphism with the risk and severity of hypospadias.

**Methods:**

263 congenital hypospadias children and 312 healthy children were recruited. The polymorphism of* RYR1 *C6487T in the peripheral blood was detected by polymerase chain reaction-restriction fragment length polymorphism, and different genotypes and allelic genes were analyzed to explore their associations with the risk of congenital hypospadias.

**Results:**

The distribution frequencies of CC/CT/TT genotypes and two alleles (C and T) at* RYR1 *C6487T showed significant differences between the case and control groups (*P *< 0.05). The frequency of C allele in the case and control groups was 46.95% and 54.94%, respectively, and of T allele was 53.05% and 45.06% (*P *< 0.05). In addition, the distribution frequency of CC/CT/TT genotypes exhibited significant difference between patients with mild hypospadias and those with moderate or severe hypospadias (all* P *> 0.05), suggesting that* RYR1 *C6487T polymorphism is correlated with the severity of congenital hypospadias (X^2^ = 13.722,* P *= 0.001).

**Conclusion:**

Our study demonstrated that* RYR1 *C6487T polymorphism might be associated with an increased risk of congenital hypospadias in Chinese Han children. Our findings highlight the heterogeneous nature of hypospadias genetic susceptibility.

## 1. Introduction

Hypospadias is the most prevalent urogenital birth defect, which affects one in 300 live male births across the world [1]. Hypospadias involves a malformation in which the urethral meatus is located on the ventral side of the penis proximal to the tip of the glans, from the balanopreputial sulcus to the perineal area. Interestingly, hypospadias consists of distal hypospadias (58.4%), glanular hypospadias (20.8%), and proximal (20.8%) hypospadias [2]. There exists a putatively increasing incidence of hypospadias in China, the USA, Europe, and Australia [3]. Specifically, increasing trends and differences of hypospadias prevalence by urban-rural classification and geographical location in China have been noted [4]. Hypospadias may be classified as simple (glanular or penile) or severe (scrotal or perineal) based on the anatomical location of the urethral meatus. Although the pathogenesis remains largely unknown, hypospadias has been regarded as a complex disorder with both genetic and environmental contributions. Potential factors contributing to the occurrence of hypospadias include low birth weight, maternal age, exposure to diethylstilbestrol, fertility treatment, paternal subfertility, prescriptive drug use, maternal obesity, etc. [5].

Recent studies found that gene polymorphism and abnormal expression of certain genes are essential in the pathogenesis of hypospadias [6]. Formation of the male urethra during the first trimester of gestation is fully androgen dependent and, therefore, it appears reasonable to attribute the occurrence of hypospadias to the dysfunction of the androgen metabolic pathway [7]. Genetic polymorphisms in genes controlling action of androgen and biosynthesis of testosterone and dihydrotestosterone are likely important in the etiology of hypospadias [7]. Single-nucleotide polymorphism (SNP) in several genes that contribute to genital tubercle and early urethral development was associated with hypospadias risk, including BMP7, FGF10, GLI transcription factors, SHH, and WT1 [8].

The ryanodine receptors (RYRs), as the largest known ion channels, are high-conductance intercellular Ca^2+^ channels which are significant in the excitation-contraction coupling of skeletal muscles along with cardiac muscles. Ryanodine receptor type 1 (RYR1), RYR2, and RYR3 are three mammalian isoforms sharing about 70% sequence identity. The RYR1 and RYR2 are mainly expressed in skeletal and cardiac muscles, while RYR3 is originally identified in the brain [9]. RYR1 is a homotetrameric Ca^2+^ release channel with the location in the sarcoplasmic reticulum of skeletal muscle, which works to facilitate the initiation of skeletal muscle contraction [10]. The* RYR1* gene, which is 160 kb in length, has a minimum of 30 bp of intron sequence flanking each splice junction and contains 106 exons. The Ca^2+^ release channel is responsible for being encoded, and the muscle contraction is initiated by the release of Ca^2+^ and the relaxation is achieved by the rapid reuptake of Ca^2+^ by Ca^2+^ pump [11].* RYR1 *encodes the skeletal muscle isoform RYR1 and mutation in the* RYR1* gene is associated with congenital myopathies, which form a continuous spectrum of pathological features including a severe variant with onset in utero with fetal akinesia and arthrogryposis, central core disease (CCD), and malignant hyperthermia susceptibility [11–13].

Despite the fact that hypospadias is among the most common birth defects in newborns, little is known about its etiology. In this study, we aim to explore the relationship between the C6487T polymorphism in the* RYR1* gene and the risk of congenital hypospadias among Chinese children.

## 2. Materials and Methods

### 2.1. Ethical Statement

The study protocol was approved by the Committee on the Ethics of China-Japan Union Hospital of Jilin University and informed consent was obtained from each participant.

### 2.2. Study Subjects

All of the studied subjects were from the Chinese Han population. The study subjects in the case group were 263 boys aged 1-5 (mean age: 3.25 years) who underwent urethroplasty (112 cases with coronary sulcus; 87 cases with penile type; 43 cases with penoscrotal type; 21 cases with perineum type) in China-Japan Union Hospital of Jilin University from March 2012 and October 2015. All patients were 46, XY. All cases were diagnosed with isolated hypospadias without other genital tract malformations, such as indirect hernia, cryptorchidism, and hydrocele of tunica vaginalis. Cases were classified as mild (meatus limited to the coronal or glans penis, BPA codes 752.605, 752.625), moderate (meatus on penile shaft, 752.606, 752.626), or severe (meatus at penoscrotal junction or perineal area, 752.607, 752.627) [14]. Patients with undescended testis, intersex conditions, or known endocrine abnormalities were excluded from the study. Three hundred and 12 boys aged 2-5 (mean age: 3.31 years) who received circumcision in China-Japan Union Hospital of Jilin University between March 2012 and October 2015 were selected as controls. The subjects in the control group had normal urinary meatus without any genital tract malformations. Demographic and clinical data pertaining to reproductive profile of the mother, occupation of the parents, and family history of genital abnormalities in male blood relatives were recorded for all of the studied subjects. Besides, data of premature delivery, low birth weight, ectopic pregnancy, intake of drugs in pregnancy, and delivery times were collected.

### 2.3. Sample Collection

Two tubes of peripheral venous blood were drawn, respectively, from subjects in the case group and the control group with 5 ml in each tube and then preserved in vacuumed tubes containing ethylene diamine tetraacetic acid (EDTA) at -80°C. One tube was added with EDTA anticoagulant and reserved at -80°C for the extraction of genomic DNA. The other tube, without anticoagulant, was centrifuged at the rate of 1400 X g for 10 min to isolate the serum and then reserved at - 80°C for further experiments. The blood sample with anticoagulant was used to extract genomic DNA through whole blood genomic DNA kit (Tiangen Biotech Co., Ltd., Beijing, China) and the content was examined by ultraviolet spectrophotometer.

### 2.4. Single-Nucleotide Polymorphism (SNP) Sequencing

The polymerase chain reaction-restriction fragment length polymorphism (PCR-RFLP) was employed to test the polymorphism of* RYR1* C6487T. According to the data available in Hapmap database (http://www.hapmap.org) about RYR1 polymorphism in Chinese population, RYR1 gene is found to be 106 kb in length, containing 106 exons and encoding 5324 amino acids. The 6487th bp of RYR1 gene is present in exon 39 which is 253 bp in length, encoding amino acid residues 2110-2183. The primer of C6487t exon was designed through primer 5 offered by Sinogenomax Co., Ltd. (Beijing, China). Primer sequences were as follows: forward primer: 5'-TGC TCG AGT GCC TCG GCC AGAT-3'; reverse primer: 5'-TTC TCC TCC TGG GGG CCC ATCT-3'. Primers were custom-synthesized by Synbio Technologies (Suzhou, China).

PCR reaction system (20 *μ*l) included 2*μ*l of 10 X buffer, 1*μ*l of forward and reverse primer (5 pmol/*μ*l), respectively, 0.5*μ*l of 10 mmol/l dNTP, 0.5*μ*l of Taq polymerase (Takara Shuzo Co. Ltd., Tokyo, 5 U/*μ*l), and 2*μ*l of template (25 ng/*μ*l ul), with the addition of double distilled water to 20*μ*l. PCR reaction condition included initial denaturation at 95°C for 5 min, denaturation at 95°C for 30s, annealing at 54°C for 30s, and extension at 72°C for 40s, together with 35 cycles with the total extension at 72°C for 10 min. During each PCR reaction process, a negative control was set. Namely, we substituted templates with sterile double distilled water to avoid pollution in the PCR reaction. The mixture of 3*μ*l of PCR products and 6 × loading buffer undergone 3% agarose gel electrophoresis under 100 V for 15 min, and then the electrophoresis result was observed through gel imaging system after ethidium bromide (EB) staining. PCR product enzymatic system (15*μ*l) included 5*μ*l of PCR product, 1.5 *μ*l of 10 X enzyme buffer, 0.5 *μ*l of special restriction endonuclease PfIFI (NEB Co., Ltd., USA, 10 U/*μ*l), and the addition of sterile double distilled water to 15*μ*l. The reaction was ended 16 h after water bath and enzyme digestion at 37°C. Then the product underwent 3% agarose gel electrophoresis and hematoxylin-eosin (HE) staining, and the gel imaging system was used to observe the result. Similarly, negative control was set up in each enzyme digestion reaction to ensure the accuracy. PCR product was sent to test the sequence after electrophoresis, recovery, and purification. The ABI 3100 Sequencer and dideoxy-chain were adopted in sequence analysis, and the process was done by Sinogenomax Co., Ltd. (Beijing, China).

### 2.5. Statistics

Measurement data was presented as mean ± standard deviation (SD), and enumeration data was exhibited as percentage and ratio. The Statistical Program for Social Sciences (SPSS) 21.0 software (SPSS, IBM, West Grove, PA, USA) was used for data analysis. Chi-square test was adopted to test whether the distribution of genotypes matched with Hardy-Weinberg and to compare the difference in the distribution of the genotypes and alleles. All tests were two-tailed, with the level of significance set to* P* < 0.05.

## 3. Results

### 3.1. Ectopic Pregnancy, Pregnancy Cycle, and Fetal Weight Are Associated with the Incidence of Congenital Hypospadias

The baseline characteristics of 263 male patients with congenital hypospadias and 312 male patients who received circumcision are shown in Table 1. The case numbers of preterm delivery, ectopic pregnancy, and low birth weight in the case and control groups were 64 versus 20, 75 versus 26, and 86 versus 39, respectively, indicating that the proportions of patients with incidence of premature delivery, low birth weight, and ectopic pregnancy in the case group were significantly higher than those in the control group (all* P* < 0.05), but there were no statistic differences in the intake of drugs in pregnancy, maternal age, and delivery times between two groups, with the case numbers of 43 versus 39, 27.30 ± 3.48 versus 26.91 ± 5.70, and 1.66 ± 0.70 versus 1.71 ± 0.51, respectively (all* P* > 0.05), suggesting that ectopic pregnancy, pregnancy cycle, and fetal weight affected the incidence of congenital hypospadias.

### 3.2. Mutation of C to T Occurs in the C6487T of RYR1 Gene

**Figure 1:**
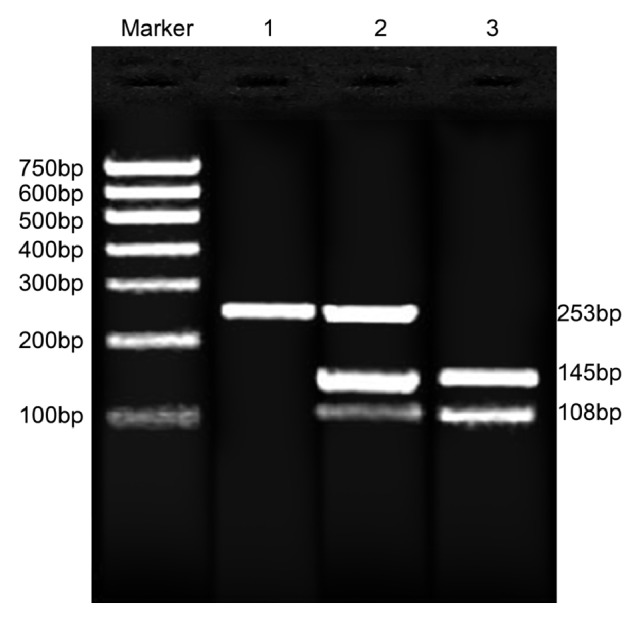
Electrophoresis diagram of PCR products. Polymerase chain reaction-restriction fragment length polymorphism (PCR-RFLP) was employed to test the* RYR1* C6487T polymorphism. The electrophoresis results of PCR amplification of target gene fragment were exhibited. The wild-type homozygote CC without restriction site yielded a fragment of 253 bp in length, heterozygote CT yielded three fragments of 253 bp, 108 bp, and 145 bp in length, and mutant homozygote TT yielded two fragments of 108 bp and 145 bp in length. PCR products performed SNP sequencing after electrophoresis, recovery, and purification. M: DNA marker; 1, wild-type homozygote; 2, heterozygote; 3, mutant homozygote; PCR, polymerase chain reaction.

**Figure 2:**
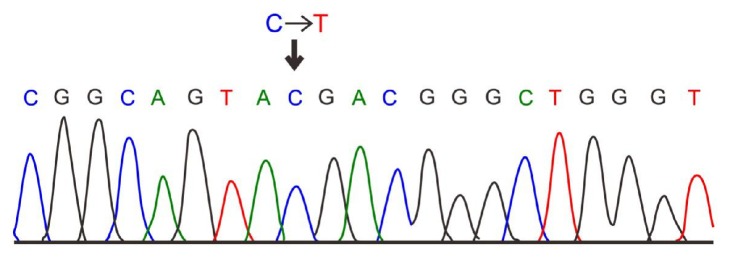
Sequencing diagram of PCR products. The sequence diagram was displayed. The C→T mutation on the C6487T in the* RYR1* gene led to the amino acid substitution: Arg2163→Cys. PCR, polymerase chain reaction.

The electrophoresis results of PCR amplification of target gene fragment were exhibited in Figure 1. The wild-type homozygote CC without restriction site yielded a fragment of 253 bp length, heterozygote CT yielded three fragments of 253 bp, 108 bp, and 145 bp length, and mutant homozygote TT yielded two fragments of 108 bp and 145 bp length. After electrophoresis, PCR products were gel purified and SNP was genotyped. The sequence diagram was displayed in the Figure 2. The C→T mutation on the C6487T in the* RYR1* gene led to the amino acid substitution: Arg2163→Cys.

### 3.3. Significantly Different Genotype and Allele Frequency Distribution of C6487T Polymorphism

**Figure 3:**
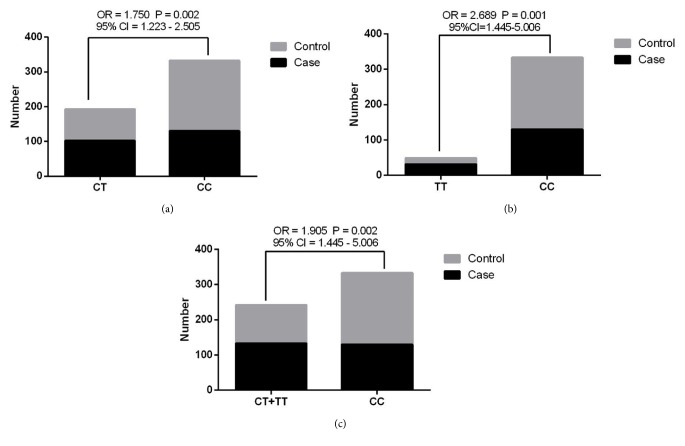
Genotype and allele frequency distribution of C6487T polymorphism of* RYR1* gene between the case and control groups. The frequency distribution of CT, TT, and CT + TT genotypes of* RYR1 *C6487T polymorphism between the case and control groups exhibited significant differences, as well as the frequency distribution of T allele (CT versus CC: OR = 1.750,* P* = 0.002, and 95% CI = 1.223-2.505; TT versus CC: OR = 2.689,* P *= 0.001; CT + TT versus CC: OR = 1.905,* P* = 0.002, and 95% CI = 1.445-5.006; T versus C: OR = 1.773,* P* < 0.001, and 95% CI = 1.356-2.319) (all* P* < 0.05).

Genotype and allele frequency distributions of* RYR1* C6487T polymorphism were tested by Hardy-Weinberg equilibrium, and all reached genetic equilibrium with group representativeness (all* P* > 0.05). As shown in Table 2 and Figure 3, the frequency distributions of CC, CT, TT, and CT + TT genotypes in the case and control groups were 130 versus 203, 102 versus 91, 31 versus 18, and 133 versus 109 with the proportions of 49.43% versus 65.06%, 38.78% versus 29.17%, 11.79% versus 5.77%, and 50.57 % versus 34.94%, respectively. The frequency distributions of T and C alleles in the case and control groups were 164 versus 127 and 362 versus 497 with the proportions of 31.18% versus 20.35% and 68.82 % versus 79.65%, respectively. The frequency distribution of CT, TT, and CT + TT genotypes of* RYR1 *C6487T polymorphism between the case and control groups exhibited significant differences, as well as the frequency distribution of T allele (CT versus CC: OR = 1.750,* P* = 0.002, and 95% CI = 1.223-2.505; TT versus CC: OR = 2.689,* P *= 0.001; CT + TT versus CC: OR = 1.905,* P* = 0.002, and 95% CI = 1.445-5.006; T versus C: OR = 1.773,* P* < 0.001, and 95% CI = 1.356-2.319) (all* P* < 0.05). The frequency distribution of CC genotype and C allele between the case and control groups was not significantly different. The results indicated patients carrying CT, TT genotypes, and T allele of* RYR1 *C6487T polymorphism may increase the incidence of congenital hypospadias.

### 3.4. C6487T Polymorphism Correlates with the Severity of Congenital Hypospadias

**Figure 4:**
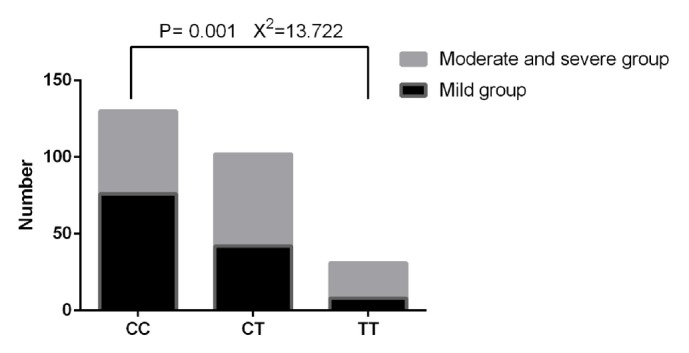
Genotype and allele frequency distribution of C6487T polymorphism of* RYR1* gene between the mild group and the moderate and severe group. The case numbers of CC, CT, and TT genotypes were 76, 42, and 8 in the mild group with the proportions of 60.32%, 33.33%, and 6.35%, respectively, while those in the moderate and severe group were 54, 60, and 23 with the proportions of 39.42%, 43.80%, and 16.79%, and the frequency distributions between two groups were significantly different (X^2^ = 13.722,* P* = 0.001).

According to the severity difference of the disease, 263 patients were distributed into two groups, i.e., the mild group (anterior segment, n = 126) and the moderate/severe group (middle and posterior segments, n = 137). The case numbers of CC, CT, and TT genotypes were 76, 42, and 8 in the mild group with the proportions of 60.32%, 33.33%, and 6.35%, respectively (Table 3 and Figure 4). The case numbers of CC, CT, and TT genotypes in the moderate/severe group were 54, 60, and 23 with the proportions of 39.42%, 43.80%, and 16.79% (Table 3 and Figure 4), and the frequency distributions between two groups were significantly different (X^2^ = 13.722,* P* = 0.001). No significant difference was seen between the moderate group and the severe group (*P* > 0.05). These results suggested that* RYR1* C6487T polymorphism may be associated with disease severity in patients with congenital hypospadias and that CT and TT genotypes may aggravate the severity of congenital hypospadias. When analyzing the risk of hypospadias with respect to phenotypic severity, we observed a significant difference in distribution of CT and TT genotypes between cases of severe phenotype and controls, further validating that CT and TT genotypes may aggravate the severity of congenital hypospadias (stratified analysis data not shown due to the relatively small sample size).

### 3.5. C6487T Polymorphism Is an Independent Risk Factor of Congenital Hypospadias

With congenital hypospadias as the dependent variable and premature birth, ectopic pregnancy, low birth weight, and* RYR1* C6487T served as independent variables for logistic regression analysis to identify the risk factors of congenital hypospadias. Low birth weight and* RYR1* C6487T were independent risk factors of congenital hypospadias (all* P* < 0.05), among which low birth weight increased the risk of congenital hypospadias by 2.755 times (*P* = 0.020, OR = 2.755, and 95% CI = 1.169-6.492), and* RYR1 *C6487T polymorphism increased the risk of congenital hypospadias by 2.982 times (*P* < 0.001, OR = 2.982, and 95% CI = 2.178-4.084). There was no statistical significance observed in premature birth (*P* = 0.327, OR = 1.745, and 95% CI = 0.573-5.319) and ectopic pregnancy (*P* = 0.773, OR = 0.236, and 95% CI = 0.603-7.780) (all* P *> 0.05) (Table 4).

## 4. Discussion

Congenital hypospadias has been the most challenging field of pediatrics with increasing prevalence worldwide, yielding complicated classifications and high incidence of postoperative complications [15–17]. We measured the polymorphism of* RYR1 *C6487T by using polymerase chain reaction-restriction fragment length polymorphism and analyzed different genotypes and allelic genes to explore their associations with the risk of congenital hypospadias. Our results displayed that* RYR1 *C6487T polymorphism may be associated with the susceptibility to congenital hypospadias.

Initially, our findings showed that the proportions of patients with congenital hypospadias in premature, low birth weight infants and gestational abnormality were significantly higher than those in the control group. To our knowledge, genetic factors have been reported to be highly involved in the etiology of hypospadias in some studies, including gene mutation that contributes to hypospadias in the presence of some genes being important for the development of the urogenital system [18]. Interestingly, a variety of environmental factors and risk of hypospadias were explored according to Zanden et al.; pregnancy complications, maternal drug use, low birth weight, and other environmental factors were regarded as key reasons for increasing the risk of hypospadias [3].

More importantly, we also found that the distribution frequencies of CC/CT/TT genotypes and two alleles (C and T) at* RYR1 *C6487T polymorphism showed statistical significance between two groups, suggesting that* RYR1 *C6487T polymorphism may contribute to the susceptibility to hypospadias.* RYR1 *gene was extensively expressed in the skeletal muscle that was involved in the release of Ca^2+^channel from the SR and excitation-contraction coupling of the skeletal muscle, thus resulting in muscle contraction [19]. Additionally, mutations in the* RYR1 *gene led to some skeletal muscle disorders and multiple diseases that are characterized by malignant hyperthermia and central core disease belonging to congenital disease.* RYR1 *variants are presented to influence RYR1 Ca^2+^release that led to sarcoplasmic reticulum Ca^2+^ leak and Ca^2+^ depletion, which causes muscle weakness [20]. Moreover,* RYR1 *mutations may produce uncontrolled Ca^2+^release that is expected to cause continuous muscle contractions, increased core temperature, and even death [21]. In pig populations,* RYR1* gene polymorphisms (C/C and C/T genotypes) significantly influence muscle growth and meat quality. More importantly, the C allele at the* RYR1* gene is highly associated with osteochondral lesions [22, 23]. Another study confirmed that different genotypes of* RYR1* gene mutations may lead to two kinds of skeletal muscle genetic diseases: malignant hyperthermia and central core disease, both of which are autosomal dominant genetic diseases [24–26].

Currently, researchers have attempted to reveal the occurrence of congenital hypospadias from the molecular level; hence more genes and their mutations are investigated to confirm their associations with hypospadias [27–29]. Collectively, we innovatively explored the relationship between* RYR1 *C6487T polymorphisms and congenital hypospadias and further investigated more valuable diagnostic indicators to provide possible therapies for the disease.

Our study has limitations. The sample size is relatively small and limits the statistical power of data analysis. As a result, stratified analyses were infeasible. Also, association analysis between risk factors, e.g., environmental exposures and hypospadias in a multivariable model, was not performed due to insufficiency of data. Underascertainment of mild cases and misclassification of severity are potential limitations as well. More importantly, we cannot clarify how the function of RYR1 is linked to the development of hypospadias and what may be the causal variant underlying the association. However, since RYR1 was not implicated in hypospadias etiology before, our findings are potentially important. Given the fact that replication is a major issue in genetic studies, we suggest that more prospective studies with larger sample size be conducted to confirm the effects of* RYR1 *C6487T polymorphisms on congenital hypospadias.

In summary, our study suggests that* RYR1 *C6487T polymorphism may contribute to the risk of congenital hypospadias. Elucidation of underlying causal variants could be useful for genetic counseling purposes or for directing mechanistic studies.

## Figures and Tables

**Table 1:** Demographics of the studied subjects.

	Case group	Control group	X^2^/t	*P*
	(n = 263) (%)	(n = 312) (%)		
Premature delivery (case)	64 (24.33)	20 (6.41)	36.75	< 0.001
Ectopic pregnancy	75 (28.52)	26 (8.33)	40.15	< 0.001
Low birth weight (case)	86 (32.70)	39 (12.50)	34.22	< 0.001
Intake of drugs in pregnancy (case)	43 (16.35)	39 (12.50)	1.73	0.388
Maternal age (age)	27.30 ± 3.48	26.91 ± 5.70	0.988	0.334
Delivery times (case)	1.66 ± 0.70	1.71± 0.51	1.526	0.128

**Table 2:** Distribution frequencies of genotypes and alleles on the C6487T in the *RYR1 *gene.

Genotypes	Case group (n = 263)	Control group (n = 312)	*P*	OR	95% CI
	(%)	(%)			
CC	130 (49.43)	203 (65.06)			Reference
CT	102 (38.78)	91 (29.17)	0.002	1.75	1.223-2.505
TT	31 (11.79)	18 (5.77)	0.001	2.689	1.445-5.006
CT + TT	133 (50.57)	109 (34.94)	0.002	1.905	1.362-2.665
C allele	362 (68.82)	497 (79.65)			Reference
T allele	164 (31.18)	127 (20.35)	< 0.001	1.773	1.356-2.319

OR, odds ratio; CI, confidence intervals.

**Table 3:** Relationship between C6487T polymorphism in the *RYR1* gene and congenital hypospadias.

Genotypes	CC	CT	TT	X^2^	*P*
Mild group (n = 126)	76 (60.32)	42 (33.33)	8 (6.35)	13.722	0.001
Moderate/severe groups (n = 137)	54 (39.42)	60 (43.80)	23 (16.79)		

**Table 4:** Logistic regression analysis of related risk factors of congenital hypospadias.

Factor	B	*P*	OR	95% CI
Premature delivery	0.557	0.327	1.745	0.573-5.319
Ectopic pregnancy	0.773	0.236	2.167	0.603-7.780
Low-birth weight	1.014	0.02	2.755	1.169-6.492
*RYR1* C6487T	1.093	< 0.001	2.982	2.178-4.084

OR, odds ratio; CI, confidence intervals.

## Data Availability

All raw data are available at request.
